# Metabolomics Characterization of Chemical Composition and Bioactivity of Highland Barley Monascus Tea Decoction Before and After Simulated Digestion In Vitro

**DOI:** 10.3390/foods13233950

**Published:** 2024-12-06

**Authors:** Haiyu Wu, Bin Dang, Wengang Zhang, Jie Zhang, Wancai Zheng, Jing Hao, Ping Ma, Xijuan Yang

**Affiliations:** 1 College of Agriculture and Animal Husbandry, Qinghai University, Xining 810000, China; wuhaiyu8828@163.com; 2 Key Laboratory of Agricultural Product Processing on Qinghai-Tibetan Plateau, College of Agricultural and Forestry Sciences, Qinghai University, Xining 810000, China; 2008990019@qhu.edu.cn (B.D.); 2017990098@qhu.edu.cn (W.Z.); 2015990070@qhu.edu.cn (J.Z.); 13565849218@163.com (W.Z.); 3 Laboratory of Qinghai-Tibetan Plateau Germplasm Resources Research and Utilization, Qinghai Academy of Agricultural and Forestry Sciences, Xining 810000, China; 4 Qinghai Tianyoude Science and Technology Investment Management Group Co., Qinghai Engineering Research Center for Comprehensive Utilization of Qinghai Highland Barley Resources, Xining 810000, China; haojing.27@163.com (J.H.); mapingyz@163.com (P.M.)

**Keywords:** highland barley Monascus tea decoction, digestion in vitro, metabolomics characterization, activity

## Abstract

A broadly targeted metabolomics approach based on UPLC-MS/MS was employed to investigate the changes in chemical composition and in vitro activity of highland barley Monascus tea decoction before and after simulated digestion. The characteristic metabolites of the tea decoction before and after in vitro-simulated digestion were identified, and the in vitro antioxidant and enzyme inhibitory activities of the tea decoction were further analyzed. The study detected 1431 metabolites, including amino acids and their derivatives, alkaloids, organic acids, nucleotides and their derivatives, lipids, terpenoids, and phenolic acids. A total of 136 differential compounds were identified, primarily distributed in amino acids and their derivatives, alkaloids, organic acids, phenolics, and lipids. in vitro-simulated digestion significantly increased the content of amino acids, alkaloids, lipids, and phenolics in the tea. The differential metabolic compounds were primarily assigned to 20 metabolic pathways, mainly involving the metabolism of amino acids, nucleotides, carbohydrates, fatty acids, and other compounds. Additionally, after simulated digestion in vitro, the comprehensive antioxidant index (60.53%), α-glucosidase inhibitory activity (54.35%), and pancreatic lipase inhibitory activity (4.06%) was significantly improved. The highland barley Monascus tea decoction showed potential hypoglycemic and hypolipidemic efficacy. This study can provide a theoretical basis for the high-value utilization of highland barley and the development of healthy grain tea.

## 1. Introduction

Highland barley, also known as naked barley, is a fundamental crop primarily cultivated on the Qinghai–Tibet Plateau. It is characterized by its nutritional properties known as “three high and two low”: high levels of protein, dietary fiber, and vitamins, along with low sugar and fat content [1]. Highland barley surpasses corn and rice in its abundance of β-glucan, polyphenol, tocopherol, and other functional factors, offering benefits such as antioxidant, anti-tumor [2], hypoglycemic [3], anti-cardiovascular disease, and immunity enhancement [4]. Highland barley Monascus tea is a kind of barley-fermented tea. Its production process involves washing the highland barley and then high-temperature sterilization, cooling down after the inoculation of Monascus for 18 days of solid-state fermentation, and finally freeze-drying to get the highland barley Monascus tea. Highland barley Monascus tea offers enhanced functional components and distinctive sensory attributes compared to highland barley. These qualities position it as an emerging functional barley tea product gaining popularity in the market.

It is well-known that food-active ingredients undergo dynamic changes during digestion, and many factors affect their utilization and biological functions in the body. These factors include the stability of substances during gastrointestinal digestion, their release from macromolecular components, their interactions with other food elements, and their absorption through the small intestine’s cells, which all influence the bioavailability of these active ingredients [5,6]. Although highland barley Monascus tea is a newly developed functional product made from biologically processed highland barley, there is limited detailed information on its functional properties and how they change during digestion. This lack of information hinders our understanding of the potential health benefits of highland barley Monascus tea. In vitro-simulated gastrointestinal digestion serves as a practical, rapid, and economical method to replicate the food-digestion and absorption process in the human body. This technique is extensively employed to investigate the digestion and absorption of food-active ingredients and the influence of processing technologies on the bioavailability of these components [7,8]. Glahn et al. demonstrated that the bioavailability of iron ions, as assessed through an in vitro digestion model, aligns with outcomes from human studies [9]. Wu et al. used simulated in vitro gastrointestinal digestion to investigate the stability, antioxidant activity, and in vitro bile acid binding of tea polyphenols from green, black, and dark teas during simulated digestion [10]. This finding highlights the significance of employing in vitro-simulated digestion to study the impact on the active ingredients of highland barley Monascus tea, which is essential for its scientific evaluation and informed consumption.

In recent years, metabolomics technology based on ultra-high-performance liquid chromatography-tandem mass spectrometry (UPLC-MS/MS) has been widely utilized for the analysis and identification of plant compounds due to its advantages of high throughput, rapid separation, high sensitivity, and broad coverage [11]. This technique serves as a potent tool for investigating the functional components within highland barley Monascus tea decoction and the bioactive substances present in the tea soup. Considering the undefined chemical composition, digestive traits, and biological activities of highland barley Monascus tea decoction, an extensive targeted metabolomics approach was employed to characterize the primary components and various compounds within the decoction before and after in vitro-simulated digestion. The study further investigated the antioxidant, amylase inhibitory, and lipase inhibitory activities of the tea soup in vitro. It also explored the association between the alterations in the functional components of the highland barley Monascus tea decoction and its in vitro activities before and after simulated digestion. The primary aim of the research is to establish a theoretical foundation for the advancement and application of functional fermented tea derived from highland barley.

## 2. Materials and Methods

### 2.1. Materials and Reagents

ACCC 30352 *Monascus purpureus*, provided by Shanghai Ruichu Biotechnology Co., LTD. Culture Preservation Center (RCCC). Highland barley was sourced from the Qinghai Academy of Agricultural and Forestry Sciences. The following chemicals were of analytical grade: ethyl acetate, glucose, sodium bicarbonate, oxalic acid, disodium hydrogen phosphate, sodium dihydrogen phosphate, sodium acetate, potassium persulfate, hydrochloric acid, sodium carbonate, ferric chloride, methanol, and phenol. Folin reagent was supplied by Beijing Solarbio Science & Technology Co., Ltd. Sigma Co. (St. Louis, MO, USA) provided 1,1-diphenyl-2-picrylhydrazyl radical (DPPH), 2,4,6-tripyridyl-s-triazine (TPTZ), 2,20-azinobis-(3-ethylbenzthiazoline-6-sulfonate) (ABTS), α-amylase (3700 U/g), α-glucosidase (26.5 U/mg), and lipase (50 U/mg). Artificial saliva, gastric juice, and intestinal fluid were obtained from Shanghai Yuanye Biotechnology Co., Ltd (Shanghai, China).

### 2.2. Sample Preparation

#### 2.2.1. Preparation of Highland Barley Monascus Tea Decoction

Mature highland barley grains were selected, washed, and soaked in distilled water for 36 h, then drained and sterilized at 121 °C for 25 min. After cooling in a sterile environment, the grains were inoculated with *Monascus purpureus*, stirred thoroughly, and incubated at an optimal temperature. The mixture was agitated every 2 days and allowed to ferment for 16 days. Subsequent freeze-drying reduced the moisture content to below 10%, yielding the final highland barley Monascus tea product. For the preparation of the tea decoction, 7.0 g of the tea sample was weighed and brewed in boiling water using a 1:28 g/mL material-to-liquid ratio. After 5 min of brewing and subsequent filtration, the decoction was cooled to obtain the highland barley Monascus tea decoction. 

#### 2.2.2. Simulated Digestion In Vitro

In simulated oral digestion (SD), 5 mL of tea decoction was mixed with 0.5 mL of artificial saliva. This mixture was subjected to constant temperature oscillation at 37 °C and 100 r/min for 5 min, followed by enzyme deactivation in a boiling water bath for 5 min. After centrifugation at 8000 r/min for 20 min, the supernatant was collected and stored at −20 °C as the oral digestion sample.

During simulated gastric digestion (GD), the pH of the digested fluid from SD was adjusted to 2.0 using 6 mol/L HCl. Artificial gastric juice (7.5 mL) was added, and the mixture was oscillated at 37 °C and 100 r/min for 120 min. Samples were extracted at specified intervals, subjected to enzyme inactivation in a boiling water bath for 5 min, centrifuged at 8000 r/min for 20 min, and the resulting supernatant was stored at −20 °C as the gastric digestion samples.

In simulated intestinal digestion (ID), following GD, the digestive fluid’s pH was adjusted to 7.6 using 1 mol/L NaHCO3. After adding 7.5 mL of artificial intestinal fluid, the mixture was oscillated at 37 °C and 100 r/min for 240 min. Samples were taken at designated times, treated for enzyme inactivation in a boiling water bath for 5 min, centrifuged at 8000 r/min for 20 min, and the supernatant was preserved at 20 °C as the intestinal digestion samples.

### 2.3. Non-Targeted Metabolomics Analysis

#### 2.3.1. Compound Extraction

Samples were retrieved from the −80 °C freezer and thawed until no ice remained. They were then vortexed for 10 s to ensure mixing. A 100 μL aliquot of the sample was transferred to a correspondingly numbered 1.5 mL centrifuge tube, to which 100 μL of 70% methanol containing an internal standard extract was added. The mixture was vortexed for 15 min, centrifuged at 12,000 r/min at 4° C for 3 min, and then filtered through a 0.22 μm membrane before being stored in a sample vial for UPLC-MS/MS analysis.

#### 2.3.2. UPLC-MS/MS Analysis

UPLC conditions: Agilent SB-C18 1.8 μm, 2.1 mm × 100 mm; Mobile phase: phase A is ultra-pure water (0.1% formic acid added), phase B is acetonitrile (0.1% formic acid added). The elution gradient began with 5% phase B, which linearly increased to 95% over 9.00 min and was maintained at 95% for 1 min. From 10.01 to 11.10 min, the proportion of phase B decreased back to 5% and was equilibrated at 5% until 14 min. The flow rate was set at 0.35 mL/min with a column temperature of 40 °C, and the injection volume was 2 μL.

Mass spectrum conditions: Electrospray ion source (ESI) temperature 500 °C; Ion spray voltage (IS) 5500 V (positive ion mode)/−4500 V (negative ion mode); Ion source gas I (GSI), gas II (GSII > Gas curtain gas (CUR >) was set at 50, 60, and 25 psi, respectively, and collision-induced ionization parameters were set to high. The QQQ scan was conducted in MRM mode with nitrogen as the collision gas at a medium setting. The declustering potential (DP) and collision energy (CE) for each MRM ion pair were optimized, with a specific set of MRM ion pairs monitored in each period based on the compounds eluted.

### 2.4. Determination of In Vitro Activity

#### 2.4.1. Determination of Antioxidant Activity

To assess the DPPH radical scavenging activity, 1 mL of tea decoction sample was combined with 4 mL of 0.1 mmol/L DPPH·methanol solution in a test tube. After incubating for 30 min in the dark, the absorbance was assessed at 517 nm. The scavenging ability was calculated using the standard curve (Y = −0.0042X + 0.0233, R^2^ = 0.9928), with results expressed in µmol Trolox in 100 g of sample (dry weight).

For the ABTS radical scavenging activity, 1 mL of the tea decoction sample was combined with 4 mL of ABTS scavenging working solution. After a 30-min dark incubation, absorbance was recorded at 734 nm. The scavenging ability was determined utilizing the standard curve (Y = −0.001X − 0.0118, R^2^ = 0.9907), and the results were expressed in µmol Trolox in 100 g of sample (dry weight). 

The FRAP assay involved mixing 1 mL of tea decoction sample with 4.5 mL of FRAP working solution in a test tube. After a 30-min dark incubation, absorbance at 593 nm was detected. FRAP reducing power was evaluated via the standard curve (Y = 0.0071X − 0.00118, R^2^ = 0.99921), and the results were expressed in µmol Trolox in 100 g of sample (dry weight).

Antioxidant activity composite index calculation: the antioxidant activity composite index method was used to evaluate the antioxidant capacity of highland barley Monascus tea decoction during simulated digestion in vitro.

(1) $$APC\;Composite\;Index\left(\%\right)=\frac{Measured\;values\;for\;each\;method}{The\;maximum\;value\;determined\;by\;this\;method\times The\;total\;number\;of\;method\;used}\times 100$$

#### 2.4.2. Determination of α-Amylase and α-Glucosidase Inhibitory Capacity 

To determine the α-amylase inhibition rate, a 500 μL sample of tea decoction was mixed with an equal volume of α-amylase solution (2.0 U/mL). The mixture was then incubated at 37 °C for 10 min. Subsequently, 500 μL of a 1% soluble starch solution was added, and the incubation at 37 °C continued for another 10 min. The reaction was terminated by adding 1 mL of 3,5-dinitrosalicylic acid color reagent. The mixture was then placed in a boiling water bath for 5 min, after which 10 mL of distilled water was added to dilute the solution. The absorbance was measured at a wavelength of 540 nm.

α-Amylase inhibition rate (%) = [1 − (A1 − A2)/A3] × 100%(2)

where A1 is the absorbance value of the experimental group, A2 is the absorbance value of the blank experimental group when replacing α-amylase with buffer, and A3 is the absorbance value of the control group when replacing the sample with buffer.

To assess the α-glucosidase inhibition rate, 40 μL of the tea-decoction sample and 30 μL of α-glucosidase solution (0.4 U/mL) were added to a 96-well plate, mixed, and incubated at 37 °C for 10 min. Subsequently, 30 μL of PNPG (5 mmol/L) was added to each well, mixed, and incubated again at 37 °C for 30 min. To terminate the reaction, 100 μL of Na_2_CO_3_ (1 mol/L) was added to each well. The absorbance was then measured at 405 nm.

α-glucosidase inhibition rate (%) = [1 − (A1 − A2)/A0] × 100%(3)

where A0 is the absorbance value of the blank experimental group when phosphate buffer replaces the sample, A1 is the absorbance value of the experimental group, and A2 is the absorbance value of the blank group when phosphate buffer replaces the PNPG.

#### 2.4.3. Determination of Lipase Inhibitory Capacity

To assess pancreatic lipase inhibition, 0.5 mL of lipase solution and 0.5 mL of tea decoction are mixed with 3 mL of Tris-HCl buffer (0.1 mol/L, pH 8.2) and incubated at 37 °C for 10 min. Then, 1.5 mL of p-PNL is added, and the mixture is further incubated for 2 h at 37 °C. Following incubation, the mixture is centrifuged at 16,000 rpm for 5 min. The absorbance of the supernatant is measured at 400 nm, with the buffer serving as the reference solution.

lipase inhibition rate (%) = [1 − (A1 − A2)/(A3 − A4)](4)

where A1 is the absorbance value of the experimental group, A2 is the absorbance value of the experimental blank group when Tris-HCl buffer replaces lipase, A3 is the absorbance value of the control group when Tris-HCl buffer replaces sample, and A4 is the absorbance value of the blank group when Tris-HCl buffer replaces sample and amylase.

### 2.5. Statistical Analysis 

MRM data were processed using the software Analyst 1.6.3. Substance characterization was performed based on the MWDB (Metware database) and secondary spectral information. Significant differences between means were calculated using the Student–Newman–Keuls q (SNK-q) test, with *p* < 0.05 considered statistically significant. Data analysis was conducted utilizing Excel 2010 (Microsoft, Redmond, WA, USA), and correlation images were generated using Origin 2022 (OriginLab, Northampton, MA, USA) and the Metware cloud platform (https://cloud.metware.cn/ accessed on 20 August 2023). R software (www.r-project.org/ accessed on 7 September 2023) was employed for principal component analysis (PCA) and orthogonal partial least squares discriminant analysis (OPLS-DA). Metabolic pathway analysis before and after simulated digestion in vitro was conducted using the KEGG database.

## 3. Results and Discussion

### 3.1. UPLC-MS/MS-Based Untargeted Metabolomics Analysis Results

**Figure 1:**
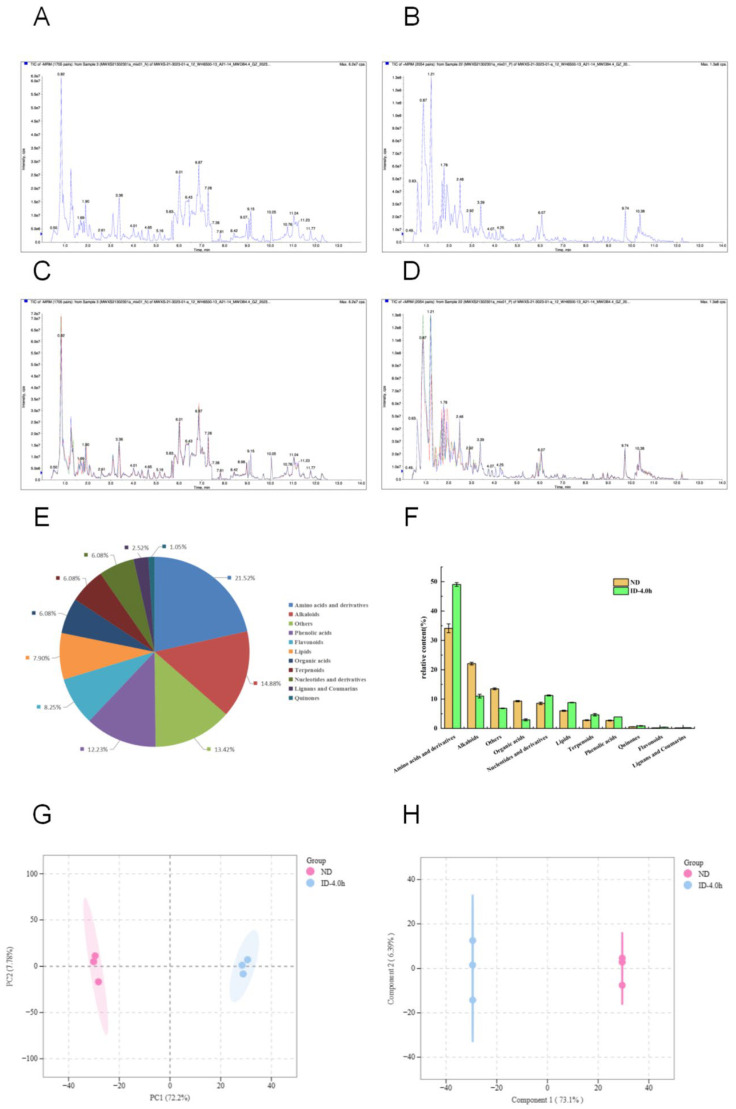
Distribution and variability of 1431 metabolites in highland barley Monascus tea decoction before and after simulated digestion in vitro. (**A**) Total ion flow plots of mixed samples and quality control (QC) samples-negative ion mode; (**B**) Total ion flow plots of mixed samples and quality control (QC) samples-positive ion mode; (**C**) MRM metabolite multi-peak detection plots-negative ion mode; (**D**) MRM metabolite multi-peak detection plots-positive ion mode; (**E**) Classifications and compositions of the 1431 metabolites; (**F**) Different classes of metabolites’ relative content; (**G**) principal component analysis of metabolites; (**H**) OPLS-DA score plot of metabolites.

In this study, 1431 metabolites were detected by UPLC-MS/MS from highland barley Monascus tea decoction before and after simulated digestion in vitro. These metabolites were classified into 11 groups: 308 amino acids and their derivatives, 213 alkaloids, 175 phenolic acids, 118 flavonoids, 113 lipids, 87 organic acids, 87 nucleotides and their derivatives, 87 terpenoids, 36 lignans and coumarins, 15 quinones, and 192 other substances. The major compounds included amino acids and their derivatives, alkaloids, organic acids, nucleotides, lipids, terpenoids, and phenolic acids, with total relative contents of 64.92% before digestion and 92.65% after digestion. The total ion flow plots (TIC, Figure 1A,B) and MRM metabolite multi-peak detection plots (Figure 1C,D) of the mixed and quality control (QC) samples demonstrated the high reproducibility and reliability of the metabolite data in this study. The principal component analysis (PCA) revealed that the first three principal components (PC1, PC2, and PC3) accounted for 87.30% of the sample information collectively, with contributions of 72.21%, 7.78%, and 7.31%, respectively, indicating that these three components represent the majority of the sample information (Figure 1G). OPLS-DA showed that the model R^2^X = 0.795, R^2^Y = 1, and Q^2^ = 0.99 > 0.9, suggesting a stable model (Figure 1H). The OPLS-DA plots indicated distinct separation between the metabolites of highland barley Monascus tea decoction before and after digestion, highlighting significant differences in the metabolites.

### 3.2. Analysis of Differential Metabolites

**Figure 2:**
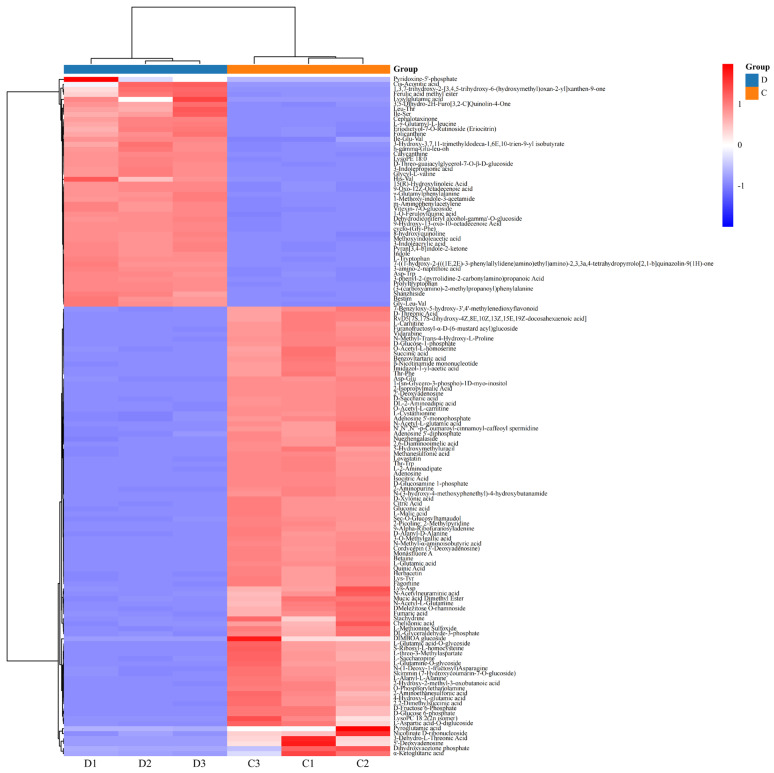
Clustering heat map of differential metabolites in highland barley Monascus tea decoction before and after simulated digestion in vitro.

**Figure 3:**
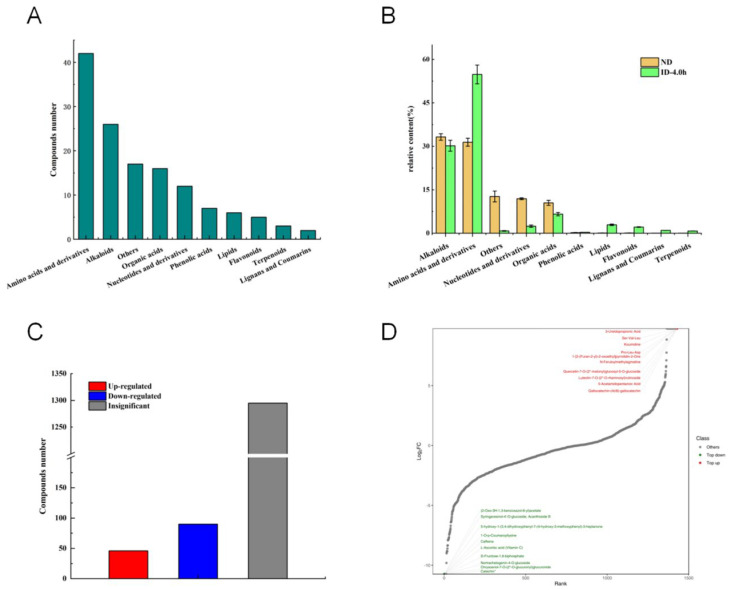
Classification and expression of different metabolites of highland barley Monascus tea decoction before and after simulated digestion in vitro. (**A**) Classification and composition of 136 differential metabolites; (**B**) the relative content of differentiated metabolites of different classes; (**C**) Up-regulated and down-regulated metabolite statistics; (**D**) Differential metabolites in the top 10 multiples of change.

A total of 136 significantly different metabolized compounds were screened in barley red tea decoction before and after in vitro-simulated digestion using |Log2FC| ≥ 4 and VIP ≥ 1 as criteria (Figure 2). These metabolites were broadly classified into 10 groups: 42 amino acids and their derivatives, 26 alkaloids, 16 organic acids, 12 nucleotides and their derivatives, seven phenolic acids, six lipids, five flavonoids, three terpenoids, two lignans and coumarins, and 17 other compounds (Figure 3A). In the undigested (ND) group, the metabolites with higher relative content were primarily alkaloids (33.21%), amino acids and their derivatives (31.40%), nucleotides and their derivatives (11.89%), and organic acids (10.48%). In contrast, the digested (ID-4.0 h) group exhibited elevated relative contents of amino acids and their derivatives (54.81%), alkaloids (30.18%), organic acids (6.58%), lipids (2.91%), nucleotides and their derivatives (2.42%), and flavonoids (2.13%) (Figure 3B). Cumulatively, 90 compounds were down-regulated, and 46 compounds were up-regulated in the digested group (ID-4.0 h group) compared to the undigested group (ND group) (Figure 3C). Figure 3D highlights the most significantly up-regulated metabolite in the digested highland barley Monascus tea decoction, which was 3-ureidopropionic acid, a breakdown product of dihydrouracil that can be further metabolized to β-alanine by β-ureidopropionase. Other notable up-regulated compounds included the peptides serinyl-valinyl-leucine and proline-leucinyl-aspartic acid, which are known for their efficient absorption and metabolism. Koumidine, an identified alkaloid, exhibits various pharmacological effects, including antitumor, anti-infective, anti-inflammatory, analgesic, and immunomodulatory activities [12,13]. Gallocatechin-(4α→8)-gallocatechin, a member of the catechin class of compounds, has been found to exhibit antimicrobial effects both in vivo and ex vivo, as demonstrated by Takabayashi F et al. [14]. It also shows a significant sensitizing effect on various antibiotics [15,16,17]. The significant increase in the relative content of these compounds after digestion may be critical for the in vivo bioactivity of barley red tea. Additionally, a down-regulation of catechin, caffeine, and ascorbic acid (vitamin C) was observed, likely due to the strong acidic conditions in the gastric digestive phase. These conditions can accelerate decomposition and oxidation reactions, leading to the degradation of these compounds and a subsequent decrease in their content.

#### 3.2.1. Amino Acids and Derivatives

Amino acids are crucial for human metabolism and vital to dietary nutrition. As shown in Table 1, 42 amino acids and their derivatives exhibited differential metabolism between the ND and ID-4.0 h groups. Compared with the ND group, 25 amino acids such as O-acetyl-L-homoserine, L-lysine-L-tyrosine, threonine-tryptophan, and L-glutamic acid were down-regulated, whereas 17 amino acids, such as γ-glutamine phenylalanine, L-leucine-L-threonine, and h-γ-glutamate-leucine -hydroxy were up-regulated in the ID-4.0 h group. In summary, the digestive enzymes destroy the structure of most amino acids and their derivatives during digestion, which promotes the release of some amino acids, but ultimately reduces the overall abundance of amino acids. Among the up-regulated amino acids, L-tryptophan, an essential amino acid, was detected, known for its role in promoting human development and enhancing immunity [18].

#### 3.2.2. Alkaloids

Alkaloids play a pivotal role in human health, offering a spectrum of biological activities, with compounds like betaine and L-carnitine regulating fat metabolism and providing antioxidant benefits. The results (Table 1) showed that 26 distinct alkaloidal compounds varied between the ND and ID-4.0 h groups. Compared with the ND group, 14 alkaloids were significantly up-regulated in the ID-4.0 h group, such as 3-indolylpropionic acid, cephalotaxinone, indole, and 8-hydroxyquinoline, while 12 alkaloids were significantly down-regulated, including imidazole-1-acetic acid, betaine, DL-2-aminoadipic acid, and L-carnitine. Overall, the in vitro-simulated digestion process proved beneficial in enhancing the dissolution and release of alkaloids in highland barley Monascus tea decoction. The reason for this is hypothesized to be mainly due to the change in pH during digestion, which may alter the binding state of the alkaloids to the food matrix and aid in their release.

#### 3.2.3. Organic Acids

Organic acids play a crucial role in the flavor profile of highland barley and significantly influence the flavor development of highland barley Monascus tea decoction. This study detected 16 different organic acid compounds in highland barley Monascus tea both before and after in vitro digestion (Table 1). Among these compounds, 15 organic acids showed a notable decrease, including 2-isopropylmalic acid, 2-aminoethanesulfonic acid, quinic acid, isocitric acid, methanesulfonic acid, succinic acid, citric acid, and fumaric acid, while only one organic acid (*cis*-aconitic acid) displayed a significant increase. The relative content of organic acids in highland barley Monascus tea decoction decreased significantly after in vitro-simulated digestion, likely due to the decomposition or transformation of these acids during digestion.

#### 3.2.4. Phenolic Substances

Phenolic compounds, known for their anti-inflammatory, antioxidant, and anticancer properties [19,20], showed significant variance between the ND and ID-4.0 h groups. A total of 14 differential phenolic compounds were identified, comprising seven phenolic acids, five flavonoids and two lignans and coumarins (Table 1). Following in vitro-simulated digestion, highland barley Monascus tea decoction exhibited significant upregulation of specific compounds, including three phenolic acids (ferulic acid methyl ester, D-threo-guaiacylglycerol-7-O-β-D-glucoside, 1-O-feruloylquinic acid), three flavonoids (1,3,7-trihydroxy-2-[3,4,5-trihydroxy-6-(hydroxymethyl)oxan-2-yl] xanthen-9-one, vitexin-7-O-glucoside, eriocitrin), and one lignan and coumarin (dehydrodiconiferyl alcohol-gamma’-O-glucoside). The enhanced phenolic content observed in highland barley Monascus tea decoction post-digestion is attributed to the breakdown of chemical bonds between phenolics and other molecules by digestive enzymes, facilitating the release of phenolics in their bound form. Increased phenolics contribute to better in vitro and in vivo highland barley Monascus tea bioactivities and its tea decoction.

#### 3.2.5. Lipids

As shown in Table 1, six differential lipid compounds were detected between the ND and ID-4.0 h groups. Among them, four unsaturated fatty acids, including 9-hydroxy-13-oxo-10-octadecenoic acid, 15(R)-hydroxylinoleic acid, 9-oxo-12Z-octadecenoic acid, and LysoPE, were significantly up-regulated in the highland barley Monascus tea decoction after in vitro digestion. These compounds are noted for their potential in promoting brain development and exhibiting anti-inflammatory properties [21]. LysoPC and RvD5[7S,17S-dihydroxy-4Z,8E, 10Z,13Z,15E,19Z-docosahexaenoic acid] showed a downregulation after in vitro digestion. Prior research has highlighted the abundance of fat digestive enzymes in the small intestine, making it the primary site for lipid digestion. This phenomenon likely contributes to the notable increase in lipid analog levels in the highland barley Monascus tea decoction following in vitro digestion [22].

### 3.3. Changes in Key Nutritional and Functional Components of Highland Barley Monascus Tea Decoction Before and After In Vitro-Simulated Digestion

**Figure 4:**
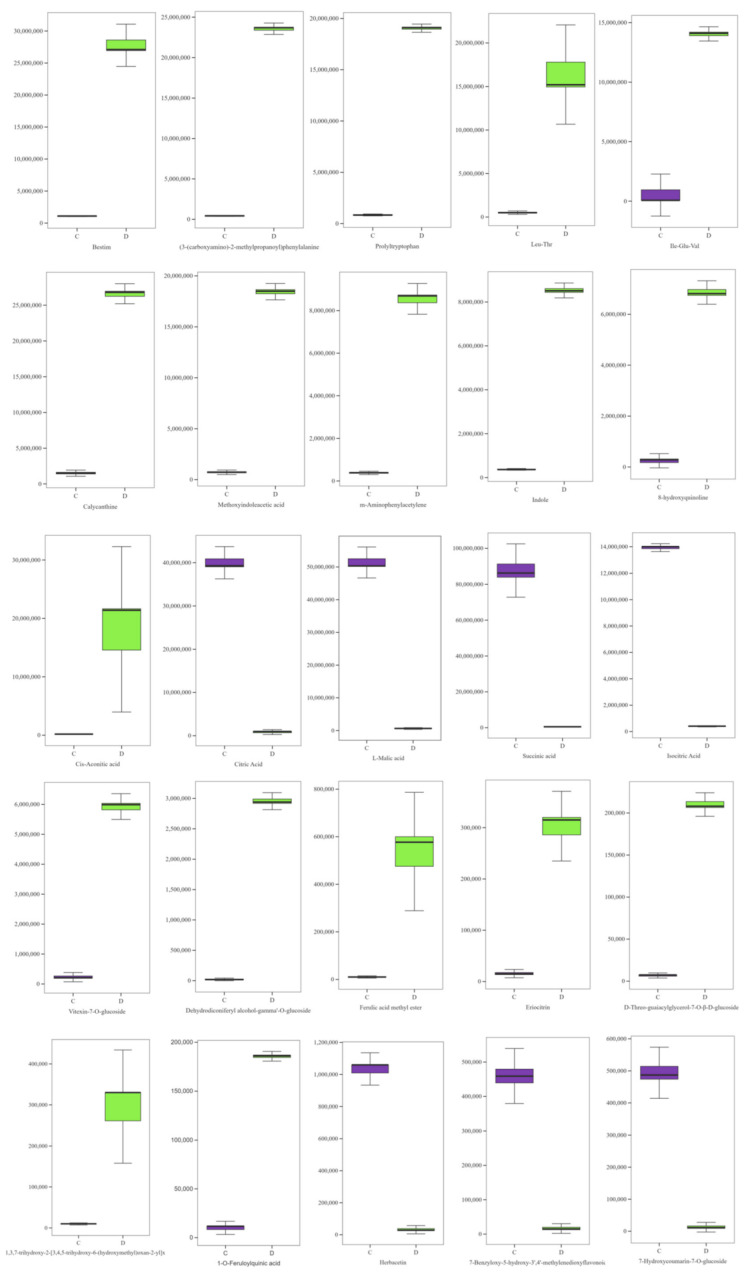
Changes in the content of five amino acids and their derivatives, five alkaloids, five organic acids, and 10 phenolic constituents in barley red tea broth before and after simulated digestion in vitro.

Significant changes were observed in the relative contents of amino acids and their derivatives, alkaloids, organic acids, and phenolics among the 10 classes of compounds in highland barley Monascus tea decoction after in vitro-simulated digestion. These components are considered important bioactive constituents of the tea decoction. Therefore, the study further analyzed the changes in five amino acids, five alkaloids, five organic acids, and 10 polyphenols, which exhibited the most significant changes in relative contents post-digestion. As shown in Figure 4, the relative contents of Bestim, (3-(carboxyamino)-2-methylpropionyl) phenylalanine, Prolyltryptophan, Leu-Thr, and Ile-Glu-Val in the highland barley Monascus tea decoction increased significantly after in vitro-simulated digestion. This increase is hypothesized to be due to the decomposition of proteins in the tea decoction into smaller peptides and amino acids with lower degrees of polymerization by various digestive enzymes during the intestinal digestion phase.

After in vitro-simulated digestion, the relative contents of five alkaloids in the tea decoction, including Calycanthine, methoxyindoleacetic acid, m-aminophenylacetylene, indole, and 8-hydroxyquinoline, were significantly increased, indicating that in vitro-simulated digestion enhances the solubilization and release of alkaloids in highland barley Monascus tea. Alkaloids often exhibit various physiological activities. For instance, Calycanthine has notable biological activities, including analgesic and antibacterial effects, particularly against *Staphylococcus aureus*. Indole helps maintain the biological barrier of the human gut and exhibits anti-inflammatory activity, primarily through the activation of AhR and PXR receptors. This activity can significantly improve gut health by influencing the function of the immune system.

The relative content of *cis*-aconitic acid increased after in vitro-simulated digestion, and the relative content of citric acid, L-malic acid, succinic acid, and isocitric acid decreased significantly. Li Q et al. [23]. reported that bound acids in the peel of jujubes could be converted into free acids under the catalytic action of various enzymes in human digestive juices, leading to an increase in organic acids during the intestinal digestion stage. However, this study’s findings differ, possibly because highland barley Monascus tea contains more free acids after Monascus fermentation. Consequently, a greater proportion of free acids may be decomposed and transformed under the action of digestive juices, resulting in a significant decrease in their relative content. The relative contents of herbacetin, 7-benzyloxy-5-hydroxy-3′, 4′-methylenedioxyflavone, and 7-hydroxycoumarin-7-o-glucoside decreased, while the contents of other phenolic compounds increased to different degrees in highland barley Monascus tea decoction after in vitro-simulated digestion. Polyphenolic compounds are known for their antioxidant, anti-inflammatory, and antiproliferative activities [24]. For example, ferulic acid methyl ester, a derivative of ferulic acid, possesses antioxidant, antibacterial, and anti-inflammatory properties [25,26]. A previous study demonstrated that ferulic acid methyl ester has higher activity, lower toxicity, and better protection against hypoxic damage to cardiomyocytes than ferulic acid [27]. 1-O-Feruloylquinic acid is a quinic acid derivative that has strong antioxidant properties and can be metabolized into tryptophan and nicotinamide by gastrointestinal flora, providing essential metabolic components for the human body. Vitexin-7-o-glucoside is an antioxidant flavonoid with biological activities such as the treatment of hyperlipidemia, fatty liver, and protection of cardiac function [28]. Eriocitrin, a common flavonoid found abundantly in citrus fruits like lemons and limes, has been shown to inhibit the proliferation of MCF7 breast cancer cells and induce apoptosis [29]. In addition, the compound has anti-inflammatory and antioxidant effects [30].

In summary, the contents of short peptides, alkaloids, *cis*-aconitic acid, and key polyphenols in highland barley Monascus tea decoction significantly increased after in vitro-simulated digestion, which may provide an important material basis for the in vitro and in vivo bioactivities exerted by highland barley Monascus tea decoction after in vitro-simulated digestion.

### 3.4. KEGG Enrichment Analysis of Differential Metabolites

**Figure 5:**
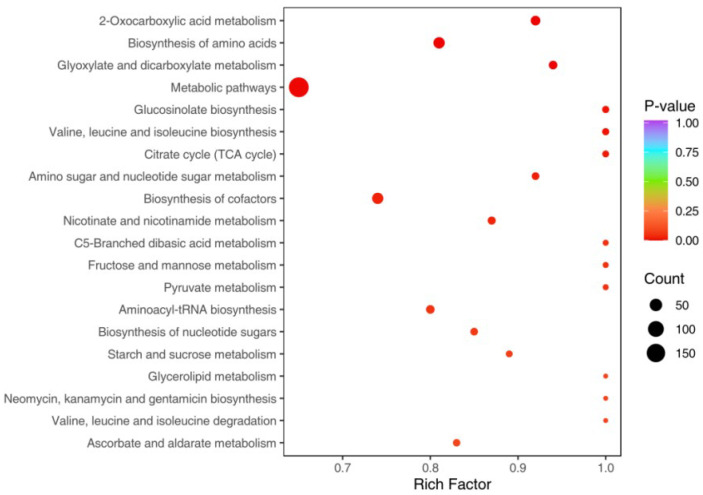
KEGG annotation and enrichment results of differential metabolites of highland barley Monascus tea decoction before and after simulated digestion in vitro.

The differential metabolites identified in highland barley Monascus tea decoction before and after in vitro-simulated digestion were analyzed using the KEGG database for pathway enrichment. Figure 5 highlights the significant impact of KEGG pathways on compound metabolism, particularly in pathways such as metabolic pathways, amino acid biosynthesis, cofactor biosynthesis, 2-oxocarboxylic acid metabolism, glyoxylate and dicarboxylate metabolism, and aminosugar and nucleotide sugar metabolism. Among these, pathways related to amino acid biosynthesis and metabolism are particularly noteworthy due to their extensive involvement in various biological reactions and metabolic processes. The differences in metabolic profiles observed in the tea decoction before and after in vitro-simulated digestion primarily involve pathways related to amino acid metabolism, indicating a pronounced influence of in vitro digestion on the amino acid composition of highland barley Monascus tea decoction. Furthermore, the distinct metabolic pathways are mainly responsible for the metabolism of amino acids, nucleotides, carbohydrates, fatty acids, and other compounds, consistent with the findings from the differential metabolite analysis.

### 3.5. Results of In Vitro Activity

#### 3.5.1. Antioxidant Activity of Highland Barley Monascus Tea Decoction Before and After In Vitro-Simulated Digestion

**Figure 6:**
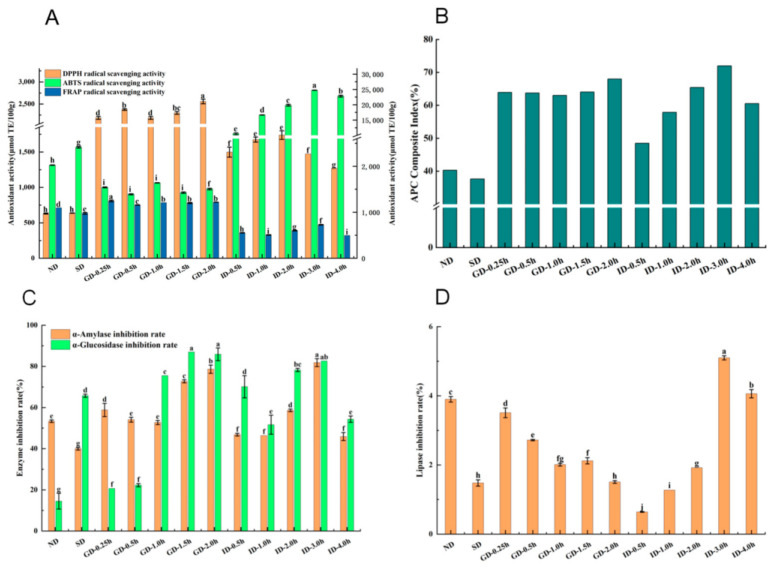
In vitro activities of highland barley Monascus tea decoction before and after simulated digestion in vitro (**A**) DPPH radical scavenging ability, ABTS radical scavenging ability and FRAP reducing ability; (**B**) APC composite index (**C**) Inhibition of α-amylase and α-glucosidase activities; (**D**) Inhibition of lipase activity. Different lowercase letters in the figure represent significant differences between groups in different digestion stages of in vitro simulated digestion for the measured indexes.

Figure 6 illustrates the antioxidant activities of highland barley Monascus tea decoction across different stages of in vitro digestion. As shown in Figure 6A, the antioxidant capacities of DPPH and FRAP in the tea decoction increased initially and then decreased during the digestion process, while the antioxidant capacity of ABTS followed the opposite trend. The DPPH radical scavenging capacity (2558.69 mg/100 g), ABTS radical scavenging capacity (24,744.90 mg/100 g), and FRAP reducing capacity (809.82 mg/100 g) reached their maximum values at GD-2.0h, ID-3.0h, and GD-0.25h, respectively. These values were 4.05, 12.22, and 3.86 times higher than those of the undigested highland barley Monascus tea decoction, respectively. The study further evaluated the changes in the antioxidant capacity of the tea decoction during the simulated digestion using the integrated antioxidant index (APC). As shown in Figure 6B, the APC of highland barley Monascus tea decoction increased significantly after in vitro-simulated digestion and reached a maximum value (71.97%) at ID-3.0h. At the end of in vitro digestion (ID-4.0 h), the APC of highland barley Monascus tea decoction reached 60.53%, 1.50 times higher than that of the ND group. Metabolomics analysis suggested that compounds such as ferulic acid methyl ester, D-threo-guaiacylglycerol-7-O-β-D-glucoside, 1-O-feruloylquinic acid, vitexin-7-O-glucoside, eriocitrin, and dehydrodiconiferyl alcohol-gamma’-O-glucoside, are likely the primary contributors to the enhanced antioxidant capacity observed post-digestion. In conclusion, phenolic compounds play a crucial role in the antioxidant activity of highland barley Monascus tea decoction, with the digestive process further enhancing its antioxidant potential.

#### 3.5.2. Inhibition of α-Amylase and α-Glucosidase Activities by Highland Barley Monascus Tea Decoction Before and After Simulated Digestion In Vitro

The inhibitory α-amylase and α-glucosidase activities of highland barley Monascus tea decoction at different digestion stages are shown in Figure 6. The inhibition of α-amylase and α-glucosidase by highland barley Monascus tea decoction ranged from 40.00–81.82% and 14.51–86.96%, respectively. Throughout the digestive process, the inhibition of α-amylase and α-glucosidase by highland barley Monascus tea decoction showed a decreasing and then increasing trend of inhibition in both gastric and intestinal digestive stages. The highest inhibition rate of α-amylase by highland barley Monascus tea decoction was observed at ID-3.0 h (81.82%), which was 1.53 times higher than that of the ND group. However, the α-amylase inhibition rate of highland barley Monascus tea decoction was significantly lower than that of the undigested group at the final stage of intestinal digestion (ID-4.0 h), potentially due to the substantial breakdown of substances with α-amylase inhibitory activity during intestinal digestion. The highest inhibition of α-glucosidase (86.96%) by highland barley Monascus tea decoction was observed at GD-1.5 h. The inhibition of α-glucosidase by highland barley Monascus tea decoction was 3.75 times higher than that of the undigested group. In the final stage of intestinal digestion (ID-4.0 h), the α-glucosidase inhibition rate of highland barley Monascus tea decoction was 3.75 times higher than that of the undigested group, indicating that highland barley Monascus tea decoction still retained a good ability to inhibit α-glucosidase after the end of digestion. Overall, while in vitro-simulated digestion had a relatively minor effect on the α-amylase inhibitory activity of highland barley Monascus tea decoction, it significantly enhanced the α-glucosidase inhibitory activity of highland barley Monascus tea decoction. Polyphenolic compounds are known to be important bioactive components for inhibiting α-amylase and α-glucosidase activities [31]. The elevated in vitro hypoglycemic capacity of highland barley Monascus tea decoction after in vitro digestion may be related to the up-regulation of many plant secondary metabolites, such as quinic acid derivatives. Studies have reported [32] that quinic acid, a natural antioxidant organic acid, has the activity of inhibiting α-amylase and α-glucosidase. Furthermore, various plant-derived compounds, such as flavonoids, polysaccharides, and free amino acids, have demonstrated inhibitory effects on α-amylase and α-glucosidase, exhibiting a mixed type of inhibition [33]. This assertion is supported by the significant up-regulation of amino acids and flavonoids observed in the tea decoction of highland barley Monascus tea after in vitro digestion (Figure 1F and Figure 2). In conclusion, the enhancement of hypoglycemic activity following in vitro digestion of highland barley Monascus tea decoction is closely associated with alterations in compounds such as phenolics, organic acids, and amino acids within the tea decoction.

#### 3.5.3. Inhibition of Lipase Activity by Highland Barley Monascus Tea Decoction Before and After Simulated Digestion In Vitro

The results of lipase inhibitory activity of highland barley Monascus tea decoction at different digestion stages are shown in Figure 6. The inhibition of lipase of highland barley Monascus tea decoction ranged from 0.64% to 5.10%. With the prolongation of in vitro-simulated digestion time, the inhibition of lipase by barley red currant tea decoction showed an overall trend of decreasing and then increasing. The highest inhibition of lipase by highland barley Monascus tea decoction was observed at 3 h of intestinal digestion (ID-3.0 h) (5.10%), which was 1.31 times higher than that of the ND group. In the final stage of intestinal digestion (ID-4.0 h), the lipase inhibition rate of highland barley Monascus tea decoction was significantly higher than that of the ND group. The elevated rate of digestive lipase inhibition in highland barley Monascus tea decoction could be attributed to the beneficial metabolism of diverse functional components present in the tea, such as phenolic acids, flavonoids, organic acids, alkaloids, and amino acids (Figure 1). However, the overall effectiveness of lipase inhibition before and after in vitro-simulated digestion by highland barley Monascus tea decoction was limited.

## 4. Conclusions

In this study, we utilized a comprehensive metabolomics approach based on UPLC-MS/MS to identify and analyze the metabolites of highland barley Monascus tea decoction before and after simulated digestion in vitro. A total of 1431 metabolites were identified and classified into 11 groups, encompassing amino acids and their derivatives, alkaloids, organic acids, nucleotides and their derivatives, lipids, terpenes, and phenolic acid metabolites. This study found that simulated in vitro digestion led to a shift in the metabolite profile of highland barley Monascus tea decoction. In vitro-simulated digestion increased the relative contents of amino acids, alkaloids, and lipids in tea decoction and promoted the release of phenolics. Furthermore, the antioxidant capacity and inhibitory activities against α-amylase, α-glucosidase, and lipase of highland barley Monascus tea decoction were observed to increase to varying degrees post-simulation, attributed to the positive metabolism of phenolic acids, flavonoids, organic acids, alkaloids, amino acids, and other functional constituents in the tea decoction. Comprehensively, the in vitro lipid-lowering effect of highland barley Monascus tea decoction is general, while its in vitro hypoglycemic potential is more prominent. This study can provide some theoretical support for the development and utilization of highland barley Monascus tea functional food.

## Figures and Tables

**Table 1:** Differential metabolites in highland barley Monascus tea decoction before and after in vitro digestion.

Compounds	Classification	CAS	VIP	*p*-Value	Fold_Change	Log_2_FC	Type
Amino acids and derivatives							
O-Acetyl-L-homoserine	Amino acids and derivatives	250736-84-6	1.16	0.00	0.06	−4.04	down
Lys-Tyr	Amino acids and derivatives	35978-98-4	1.16	0.00	0.05	−4.31	down
Thr-Trp	Amino acids and derivatives	186761-42-2	1.17	0.00	0.04	−4.64	down
L-Glutamine-O-glycoside	Amino acids and derivatives	-	1.17	0.00	0.02	−5.50	down
N-Methyl-α-aminoisobutyric acid	Amino acids and derivatives	2566-34-9	1.15	0.00	0.00	−9.81	down
L-Glutamic acid-O-glycoside	Amino acids and derivatives	-	1.17	0.01	0.00	−8.34	down
N-Acetyl-L-glutamic acid	Amino acids and derivatives	1188-37-0	1.16	0.00	0.01	−6.22	down
N-Acetyl-L-Glutamine	Amino acids and derivatives	2490-97-3	1.15	0.01	0.01	−7.18	down
Thr-Phe	Amino acids and derivatives	16875-27-7	1.17	0.00	0.06	−4.06	down
D-Alanyl-D-Alanine	Amino acids and derivatives	923-16-0	1.14	0.00	0.02	−5.50	down
Lys-Asp	Amino acids and derivatives	20556-18-7	1.16	0.01	0.04	−4.65	down
L-Alanyl-L-Alanine	Amino acids and derivatives	1948-31-8	1.17	0.00	0.03	−4.93	down
N-Methyl-Trans-4-Hydroxy-L-Proline	Amino acids and derivatives	4252-82-8	1.17	0.00	0.03	−5.07	down
L-threo-3-Methylaspartate	Amino acids and derivatives	6061-13-8	1.17	0.01	0.03	−4.84	down
L-Glutamic acid	Amino acids and derivatives	56-86-0	1.17	0.00	0.01	−7.03	down
L-Aspartic acid-O-diglucoside	Amino acids and derivatives	-	1.15	0.02	0.02	−5.50	down
Asp-Glu	Amino acids and derivatives	6157-06-8	1.15	0.00	0.06	−4.06	down
L-Cystathionine	Amino acids and derivatives	56-88-2	1.14	0.00	0.06	−4.17	down
L-2-Aminoadipate	Amino acids and derivatives	1118-90-7	1.15	0.00	0.00	−7.76	down
L-Methionine Sulfoxide	Amino acids and derivatives	3226-65-1	1.16	0.01	0.06	−4.16	down
S-Ribosyl-L-homocysteine	Amino acids and derivatives	-	1.16	0.01	0.00	−7.77	down
4-Hydroxy-L-glutamic acid	Amino acids and derivatives	3913-68-6	1.17	0.01	0.01	−6.40	down
Pyroglutamic acid	Amino acids and derivatives	4042-36-8	1.10	0.20	0.06	−4.16	down
N-Acetylneuraminic acid	Amino acids and derivatives	131-48-6	1.16	0.01	0.06	−4.12	down
L-Saccharopine	Amino acids and derivatives	997-68-2	1.13	0.01	0.04	−4.83	down
γ-Glutamylphenylalanine	Amino acids and derivatives	7432-24-8	1.17	0.00	62.87	5.97	up
Leu-Thr	Amino acids and derivatives	-	1.17	0.01	32.57	5.03	up
h-gamma-Glu-leu-oh	Amino acids and derivatives	-	1.17	0.00	17.06	4.09	up
Lysylglutamic acid	Amino acids and derivatives	45234-02-4	1.16	0.06	99.20	6.63	up
Ile-Glu-Val	Amino acids and derivatives	-	1.04	0.00	21.09	4.40	up
L-γ-Glutamyl-L-leucine	Amino acids and derivatives	2566-39-4	1.17	0.00	16.27	4.02	up
Asp-Trp	Amino acids and derivatives	71835-79-5	1.15	0.00	29.82	4.90	up
Prolyltryptophan	Amino acids and derivatives	-	1.17	0.00	22.44	4.49	up
3-phenyl-2-(pyrrolidine-2-carbonylamino)propanoic Acid	Amino acids and derivatives	-	1.17	0.00	25.46	4.67	up
Glycyl-L-valine	Amino acids and derivatives	1963-21-9	1.17	0.00	459.58	8.84	up
Bestim	Amino acids and derivatives	66471-20-3	1.17	0.00	25.95	4.70	up
(3-(carboxyamino)-2-methylpropanoyl)phenylalanine	Amino acids and derivatives	-	1.17	0.00	56.18	5.81	up
Ile-Ser	Amino acids and derivatives	6403-14-1	1.17	0.01	29.23	4.87	up
L-Tryptophan	Amino acids and derivatives	73-22-3	1.17	0.00	27.43	4.78	up
Gly-Leu-Val	Amino acids and derivatives	-	1.15	0.00	58.09	5.86	up
His-Val	Amino acids and derivatives	76019-15-3	1.16	0.02	28.10	4.81	up
cyclo-(Gly-Phe)	Amino acids and derivatives	10125-07-2	1.17	0.00	35.01	5.13	up
Alkaloids							
Compounds	Classification	CAS	VIP	*p*-value	Fold_Change	Log_2_FC	Type
Imidazol-1-yl-acetic acid	Alkaloids	22884-10-2	1.17	0.00	0.02	−5.55	down
Betaine	Alkaloids	107-43-7	1.17	0.00	0.00	−7.65	down
L-Carnitine	Alkaloids	541-15-1	1.17	0.00	0.00	−8.37	down
DL-2-Aminoadipic acid	Alkaloids	542-32-5	1.16	0.00	0.01	−7.33	down
N′,N″,N‴-p-Coumaroyl-cinnamoyl-caffeoyl spermidine	Alkaloids	-	1.17	0.00	0.03	−4.84	down
2-Picoline; 2-Methylpyridine	Alkaloids	109-06-8	1.17	0.00	0.01	−7.25	down
O-Acetyl-L-carnitine	Alkaloids	3040-38-8	1.17	0.00	0.03	−4.91	down
Stachydrine	Alkaloids	471-87-4	1.16	0.02	0.02	−5.39	down
N-(3-hydroxy-4-methoxyphenethyl)-4-hydroxybutanamide	Alkaloids	-	1.17	0.00	0.00	−7.88	down
DIMBOA glucoside	Alkaloids	18607-79-9	1.16	0.09	0.00	−9.00	down
O-Phosphorylethanolamine	Alkaloids	1071-23-4	1.17	0.00	0.03	−5.02	down
Fagomine	Alkaloids	53185-12-9	1.16	0.00	0.02	−5.95	down
3-Indolepropionic acid	Alkaloids	830-96-6	1.17	0.00	24.03	4.59	up
Cephalotaxinone	Alkaloids	38750-57-1	1.17	0.00	73.88	6.21	up
Indole	Alkaloids	120-72-9	1.17	0.00	22.60	4.50	up
8-hydroxyquinoline	Alkaloids	148-24-3	1.12	0.00	29.51	4.88	up
Methoxyindoleacetic acid	Alkaloids	3471-31-6	1.17	0.00	25.53	4.67	up
m-Aminophenylacetylene	Alkaloids	54060-30-9	1.17	0.00	22.62	4.50	up
Calycanthine	Alkaloids	595-05-1	1.17	0.00	17.79	4.15	up
7-((1-hydroxy-2-(((1E,2E)-3-phenylallylidene)amino)ethyl)amino)-2,3,3a,4-tetrahydropyrrolo [2,1-b]quinazolin-9(1H)-one	Alkaloids	-	1.16	0.00	216.50	7.76	up
Folicanthine	Alkaloids	6879-55-6	1.12	0.00	20.11	4.33	up
3-amino-2-naphthoic acid	Alkaloids	-	1.16	0.00	32.78	5.03	up
3-Indoleacrylic acid	Alkaloids	1204-06-4	1.17	0.00	29.58	4.89	up
Pyran[3,4-b]indole-2-ketone	Alkaloids	-	1.17	0.00	29.58	4.89	up
3,5-Dihydro-2H-Furo[3,2-C]Quinolin-4-One	Alkaloids	-	1.15	0.00	26.80	4.74	up
1-Methoxy-indole-3-acetamide	Alkaloids	-	1.17	0.00	37.54	5.23	up
Organic acids							
Compounds	Classification	CAS	VIP	*p*-value	Fold_Change	Log_2_FC	Type
2-Isopropylmalic Acid	Organic acids	49601-06-1	1.17	0.00	0.06	−4.14	down
2-Aminoethanesulfonic acid	Organic acids	107-35-7	1.17	0.01	0.05	−4.37	down
Quinic Acid	Organic acids	77-95-2	1.17	0.00	0.04	−4.68	down
Isocitric Acid	Organic acids	320-77-4	1.17	0.00	0.03	−5.13	down
Methanesulfonic acid	Organic acids	75-75-2	1.16	0.00	0.04	−4.58	down
Succinic acid	Organic acids	110-15-6	1.17	0.00	0.01	−7.43	down
α-Ketoglutaric acid	Organic acids	328-50-7	1.09	0.11	0.03	−4.89	down
2-Hydroxy-2-methyl-3-oxobutanoic acid	Organic acids	71698-08-3	1.16	0.00	0.02	−5.76	down
2,6-Diaminooimelic acid	Organic acids	583-93-7	1.17	0.00	0.01	−7.07	down
2,2-Dimethylsuccinic acid	Organic acids	597-43-3	1.16	0.00	0.04	−4.66	down
Citric Acid	Organic acids	77-92-9	1.16	0.00	0.02	−5.68	down
DL-Glyceraldehyde-3-phosphate	Organic acids	591-59-3	1.15	0.01	0.03	−4.98	down
Chelidonic acid	Organic acids	99-32-1	1.12	0.01	0.03	−4.95	down
L-Malic acid	Organic acids	97-67-6	1.17	0.00	0.01	−6.37	down
Fumaric acid	Organic acids	110-17-8	1.14	0.01	0.04	−4.53	down
*cis*-Aconitic acid	Organic acids	585-84-2	1.16	0.07	94.33	6.56	up
Phenolic substances							
Compounds	Classification	CAS	VIP	*p*-value	Fold_Change	Log_2_FC	Type
Furanofructosyl-α-D-(6-mustard acyl)glucoside	Phenolic acids	-	1.16	0.00	0.03	−4.84	down
Benzoyltartaric acid	Phenolic acids	-	1.17	0.00	0.04	−4.70	down
Mucic acid Dimethyl Ester	Phenolic acids	-	1.16	0.01	0.05	−4.28	down
3-O-Methylgallic acid	Phenolic acids	3934-84-7	1.16	0.00	0.00	−7.91	down
Ferulic acid methyl ester	Phenolic acids	2309-07-1	1.16	0.02	51.53	5.69	up
D-Threo-guaiacylglycerol-7-O-β-D-glucoside	Phenolic acids	-	1.16	0.00	32.39	5.02	up
1-O-Feruloylquinic acid	Phenolic acids	-	1.15	0.00	19.68	4.30	up
7-Benzyloxy-5-hydroxy-3′,4′-methylenedioxyflavonoid	Flavonoids	-	1.16	0.00	0.04	−4.79	down
Herbacetin	Flavonoids	527-95-7	1.16	0.00	0.03	−4.97	down
1,3,7-trihydroxy-2-[3,4,5-trihydroxy-6-(hydroxymethyl)oxan-2-yl]xanthen-9-one	Flavonoids	-	1.16	0.03	29.77	4.90	up
Vitexin-7-O-glucoside	Flavonoids	-	1.16	0.00	25.19	4.65	up
Eriodictyol-7-O-Rutinoside (Eriocitrin)	Flavonoids	13463-28-0	1.16	0.01	19.13	4.26	up
Dehydrodiconiferyl alcohol-gamma’-O-glucoside	Lignans and Coumarins	-	1.16	0.00	138.40	7.11	up
Skimmin (7-Hydroxycoumarin-7-O-glucoside)	Lignans and Coumarins	93-39-0	1.15	0.00	0.03	−5.23	down
Lipids							
Compounds	Classification of compounds	CAS	VIP	*p*-value	Fold_Change	Log_2_FC	Type
LysoPC 18:2(2n isomer)	Lipids	-	1.16	0.03	0.06	−4.02	down
RvD5[7S,17S-dihydroxy-4Z,8E,10Z,13Z,15E,19Z-docosahexaenoic acid]	Lipids	578008-43-2	1.17	0.00	0.04	−4.75	down
9-Hydroxy-13-oxo-10-octadecenoic Acid	Lipids	-	1.16	0.00	35.72	5.16	up
15(R)-Hydroxylinoleic Acid	Lipids	177931-23-6	1.12	0.00	39.16	5.29	up
9-Oxo-12Z-Octadecenoic acid	Lipids	112543-32-5	1.12	0.00	39.16	5.29	up
LysoPE 18:0	Lipids	69747-55-3	1.17	0.00	19.11	4.26	up

## Data Availability

The data presented in this study are available on request from the corresponding author.
